# Modeling the Effects of Vorinostat *In Vivo* Reveals both Transient and Delayed HIV Transcriptional Activation and Minimal Killing of Latently Infected Cells

**DOI:** 10.1371/journal.ppat.1005237

**Published:** 2015-10-23

**Authors:** Ruian Ke, Sharon R. Lewin, Julian H Elliott, Alan S. Perelson

**Affiliations:** 1 Theoretical Biology and Biophysics Group, Los Alamos National Laboratory, Los Alamos, New Mexico, United States of America; 2 Department of Mathematics, North Carolina State University, Raleigh, North Carolina, United States of America; 3 The Peter Doherty Institute for Infection and Immunity, the University of Melbourne, Melbourne, Australia; 4 Department of Infectious Diseases, Alfred Hospital and Monash University, Melbourne, Australia; 5 Centre for Biomedical Research, Burnet Institute, Melbourne, Australia; Massachusetts Institute of Technology, UNITED STATES

## Abstract

Recent efforts to cure human immunodeficiency virus type-1 (HIV-1) infection have focused on developing latency reversing agents as a first step to eradicate the latent reservoir. The histone deacetylase inhibitor, vorinostat, has been shown to activate HIV RNA transcription in CD4+ T-cells and alter host cell gene transcription in HIV-infected individuals on antiretroviral therapy. In order to understand how latently infected cells respond dynamically to vorinostat treatment and determine the impact of vorinostat on reservoir size *in vivo*, we have constructed viral dynamic models of latency that incorporate vorinostat treatment. We fitted these models to data collected from a recent clinical trial in which vorinostat was administered daily for 14 days to HIV-infected individuals on suppressive ART. The results show that HIV transcription is increased transiently during the first few hours or days of treatment and that there is a delay before a sustained increase of HIV transcription, whose duration varies among study participants and may depend on the long term impact of vorinostat on host gene expression. Parameter estimation suggests that in latently infected cells, HIV transcription induced by vorinostat occurs at lower levels than in productively infected cells. Furthermore, the estimated loss rate of transcriptionally induced cells remains close to baseline in most study participants, suggesting vorinostat treatment does not induce latently infected cell killing and thus reduce the latent reservoir *in vivo*.

## Author Summary

Combination antiretroviral therapy (cART) for HIV infection must be taken for life due to the existence of long lived latently infected cells. Recent efforts have focused on developing latency reversing agents to eliminate latently infected cells by activating HIV production. In this work, we assess the impact of a latency reversing agent, vorinostat, by fitting dynamic models to data from a clinical trial. Results show that vorinostat treatment induces HIV transcription transiently and that the sustained induction of HIV transcription may depend on the temporal impact of vorinostat on host gene expression. Our results also suggest that vorinostat treatment is not sufficient to induce killing of latently infected cells in a majority of HIV-infected individuals on cART.

## Introduction

Treatment of HIV-infected individuals with combination antiretroviral therapy (cART) effectively suppresses HIV to levels below the limit of detection of conventional assays and substantially reduces morbidity and mortality of HIV infected patients [1]. However, it does not eradicate the virus and treatment is lifelong [2]. Therefore, developing novel therapeutics to cure HIV infection remains an important research priority [3,4]. A major barrier to cure is the presence of a population of long lived latently infected cells [4] that can persist indefinitely in patients treated with highly potent cART [5]. Recent efforts have focused on strategies that activate HIV production in latently infected cells. The idea, termed ‘shock and kill’ [6], is to first shock latently infected cells thereby activating HIV gene expression, such that the cells are then killed by viral cytopathic effects or immune-mediated cell death. Histone acetylation is one of several factors that regulate HIV transcription and is therefore important for establishing and maintaining latency [7]. Drugs such as histone deacetylase inhibitors (HDACi) enhance acetylation of both histones and proteins and thereby induce changes in gene transcription, including transcription of HIV [8].

Vorinostat, a histone deacetylase inhibitor licensed for the treatment of cutaneous T-cell lymphoma [9], has been shown to activate HIV transcription in resting memory CD4+ T-cells *in vivo* [10,11]. In a recent clinical trial, 20 HIV-1 infected individuals on suppressive cART were treated orally with 400 mg a day of vorinostat for 14 days and then followed for an additional 70 days. Overall, vorinostat induced a rapid and sustained increase of cell-associated unspliced (CA-US) HIV RNA [10]. However, the response pattern was highly variable among the participants. For example, in half of the participants, after an initial significant increase in CA-US HIV RNA, the level of CA-US HIV RNA decreased rapidly within 1–3 days before increasing again, and in 14 of the 20 participants, the level of CA-US HIV RNA continued to increase after vorinostat was stopped. These puzzling observations raise important questions about the temporal impact of vorinostat treatment on HIV transcription and the design of treatment strategies to eradicate the latent reservoir.

We constructed mathematical models to better understand the temporal changes in CA-US HIV RNA in individuals treated with vorinostat. Mathematical models have been widely applied to study viral dynamics *in vivo* [12–14]. They played an instrumental role in quantifying important parameters, such as the half-lives of virions and infected cells *in vivo* [14]. Recently, several models have been developed to understand the maintenance of the latent reservoir under cART treatment [15–17], the viral rebound time distribution after latency reversing agent (LRA) treatment [18], and the optimal time to start a LRA [19]. However, the dynamic response of HIV transcription in latently infected cells following treatment with a LRA has not been investigated. This question has important implications for future clinical trial design and optimizing treatment strategies to eliminate latently infected cells. Previous models have generally assumed that the HIV provirus in latently infected cells becomes fully activated following treatment with a LRA and that subsequent events will be identical to latently infected cells activated by normal immunological signals or through the T-cell receptor [18,19]. However, evidence suggests that current LRA treatments primarily activate HIV transcription and its impact on translation may be mild or minimal [20,21]. Here, we construct models that treat cells activated by a LRA and naturally activated cells separately. By fitting models to the clinical data, we show the complex dynamic response of latently infected cells to vorinostat can be explained. Furthermore, we use the models to quantify the extent to which vorinostat activates HIV transcription and induces cell death *in vivo*.

## Results

### Direct Activation Model

**Fig 1:**
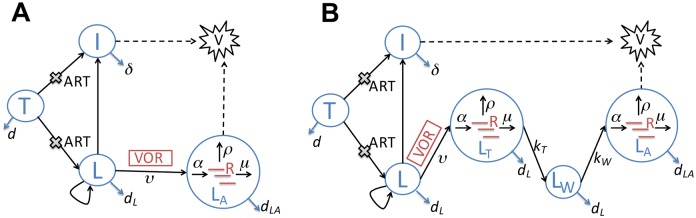
Schematic illustrations of two latency models that describe the impact of vorinostat treatment. The models keep track of both the within-host infection dynamics and intracellular HCV transactivation dynamics. **(A)** The direct activation model. CD4^+^ target cells (*T*) can be infected by HIV (*V*). Upon infection, the majority of infected target cells become productively infected cells (*I*), while a small fraction become latently infected cells (*L*). Latently infected cells (*L*) undergo asymmetric division and their progeny can either be activated or remain latent. Under vorinostat treatment, the latently infected cells become sustainably activated (*L*_*A*_) at rate *ν*. In these cells, CA-US HIV RNAs (*R*) are produced at rate *α*, exported at rate *ρ* and degraded at rate *μ*. Combination antiretroviral therapy (cART) with reverse transcriptase and protease inhibitors inhibits infection and production of infectious virus. **(B)** The delayed activation model. This model extends the direct activation model by adding two additional states: latently infected cells that are transiently activated (*L*_*T*_) upon vorinostat treatment, and cells that were transiently activated and now are in a waiting state (*L*_*W*_), i.e. a period of delay, before transitioning to a sustained activation state (*L*_*A*_). CA-US HIV RNAs (*R*) are produced from both the transiently activated cells (*L*_*T*_) and the sustainably activated cells (*L*_*A*_). Key rate constants are shown on the transitions (arrows) between compartments (see Table 1 for notation).

We first construct a mathematical model based on a previously published latency model by Rong *et al*. [15]. As the study participants were treated with suppressive cART for a medium of 5 years, we have chosen parameters in the Rong *et al*. model such that before vorinostat treatment the viral load is approximately 5 HIV RNA copies/ml, target cells levels are 750 T cells/μL similar to mean CD4 count in the 20 clinical trial subjects [10] and latently infected cell levels are 2/mL, which is approximately 2.7/million CD4 cells (roughly consistent with previous studies [22,23]). The major innovation in the direct activation model is that we assume latently infected cells become transcriptionally induced and express CA-US HIV RNA directly upon vorinostat treatment (Fig 1A; see Methods for full description of the model and Table 1 for parameter values). Thus, two equations are added to the Rong *et al*. model [15], one for the number of cells that have HIV transcription induced by vorinostat, *L*_*A*_, and one for the average amount of CA-US HIV RNA per transcriptionally activated cell, *R*. Also, cells that are transcriptionally induced by vorinostat are assumed to be in a different state than cells that are naturally activated.

**Table 1:** Description of parameters and fixed parameter values in the model.

Parameter	Description	Value	Unit	Reference
*s*	Production rate of uninfected CD4^+^ T cells	750	μL^-1^ day^-1^	
*d*	Death rate of uninfected CD4^+^ T cells	0.01	day^-1^	[24]
ε_RT_	Efficacy of reverse transcription inhibitors	0.95*		
β	Infection rate constant	2.4e-8	mL day^-1^	[25]
*f*	Probability of becoming latent upon infection	0.001		[26]
δ	Death rate of productively infected cells	1.0	day^-1^	[27]
*p*_*L*_	Probability of remaining in latency after division of a latent cell	0.55		[15]
*a*	Asymmetric division rate of latently infected cells	0.1	day^-1^	[15]
*d*_*L*_	Death rate of latently infected cells	0.01	day^-1^	[15]
*μ*	Loss rate of CA-US HIV RNA: including both degradation and splicing of CA-US HIV RNA	64.2	day^-1^	[28–30]
*ρ*	Export rate of US HIV RNA	0	day^-1^	
*ε*_*PI*_	Efficacy of protease inhibitors	0.95*		
*p*_*V*_	Production rate of HIV from productively infected cells	4000	day^-1^	[31,32]
*c*	Clearance rate of HIV	23	day^-1^	[33]
*w*	Elimination rate of vorinostat	8.31	day^-1^	[11]
*t*_*end*_	Time that treatment ends	14	days	[10]
*α*	Production rate of CA-US HIV RNA in a cell	Fitted	mL day^-1^	
*d*_*LA*_	Loss rate of transcriptionally activated cells	Fitted	day^-1^	
*ν*	Activation rate of latent cells due to the action of vorinostat treatment	Fitted	day^-1^	
*t*_*0*_	Pharmacological delay before vorinostat becomes effective	Fitted	day	
RNA_0_	US-CA HIV RNA before treatment	Fitted	molecules mL^-1^	
*k*_*T*_ (appears in the delayed activation model)	Rate of transition from the transiently activated state, L_T_, to the waiting state, L_W_.	Fitted	day^-1^	
*k*_*W*_ (appears in the delayed activation model)	Rate of transition from the waiting state, L_W_, to the sustainably activated state, L_A_.	Fitted	day^-1^	

* The parameter values for the effectiveness of the cART treatment are chosen such that the viral load is at an undetectable level (<50 copies mL^-1^) at the steady state before vorinostat treatment.

We fitted this model to the clinical data collected during the entire 84-day study period (see Methods for the fitting procedure and S1 Table for best-fit parameter values). In general, the direct activation model does not explain the data well especially during the first 1–3 days’ treatment (S1 Fig) and the period after treatment stops (S2 Fig). First, in 10 of the 20 participants, the level of CA-US HIV RNA first increased upon initiation of vorinostat, and then decreased rapidly after the first 1–3 days, whereas the best-fit model curves in these patients have CA-US HIV RNA increasing continuously during vorinostat treatment. Second, in 14 out of the 20 participants, the level of CA-US HIV RNA increased at variable time points after cessation of vorinostat at day 14, whereas the model predicts that the level always decreases over time after cessation of vorinostat.

**Fig 2:**
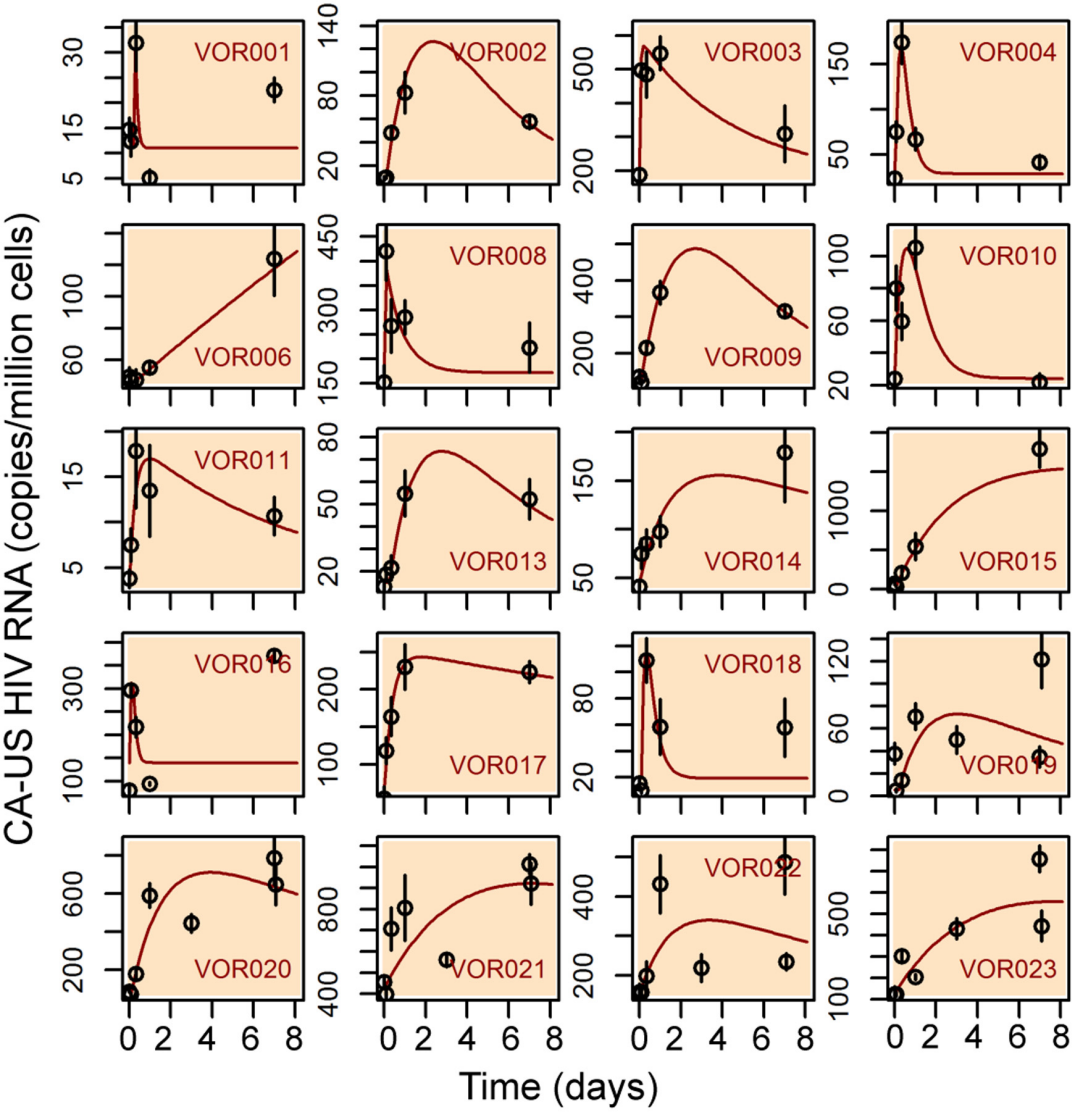
Fitting results of the direct activation model to the clinical data from the first 7-day’s of treatment. Each panel shows the fitting result for a participant. Red lines are model simulations using best-fit parameter values. The black circles and vertical black lines are the mean and standard deviation of four replicate measurements made at each time point.

To better understand the initial peaking pattern observed in half of the study participants soon after the initiation of vorinostat, we fitted the model to data obtained during the first 7 days of treatment only (Fig 2). The peaking pattern was well described by the model in five participants (VOR001, VOR004, VOR010, VOR016, VOR018). In these individuals, the estimated loss rate of transcriptionally activated cells, *d*_*LA*_, ranged between 1.8 and 10 day^-1^ (see S2 Table), and is much greater than the death rate of productively infected cells, i.e. 1.0 day^-1^ [27]. Rates of decrease of CA-US HIV RNA levels higher than 1.0 day^-1^ were also apparent in 5 other participants (VOR008, VOR019 and VOR021-023). This decrease can potentially result from either death of cells transcriptionally activated by vorinostat or from shutdown of HIV transcription and loss of CA-US HIV RNA. We reason that this decline is not due solely to cell death, as it is unlikely that cells activated by vorinostat die at a faster rate than productively infected cells. Then why would HIV transcription shut down during vorinostat treatment?

Previous *in vitro* studies have shown the activation of HIV transcription is a transient stochastic process, and that the duration of this transient process is dependent on the strength of Tat transcriptional feedback [34–36], as well as the availability and regulation of many host factors that are necessary for transcriptional activation, such as the NAD-dependent deacetylase sirtuin-1, NF-κB, Yin Yang 1 and the positive transcription elongation factor, P-TEFb [37–41]. In latently infected cells, mostly memory T cells, these transcription factors are likely to be at low levels [42–44], whereas many host enzymes such as Murr1 (a gene product that restricts HIV-1 replication), human schlafen 11 and the lipid raft associated protein tetherin, actively inhibit HIV transcription initiation [45], mRNA translation [46], and viral release [47]. Therefore, before vorinostat treatment, the host factors/enzymes required for full HIV gene activation are most likely limiting in latently infected cells. After vorinostat treatment initiation, host genes undergo rapid differential regulation at 2, 8 and 24 hours [10]; however, the immediate impact of vorinostat treatment may not be sufficient to induce HIV gene transcription sustainably. This unfavorable cellular environment and rapid changes in gene expression may lead to very short transcriptional pulses of Tat activity and CA-US HIV RNA production. Without further production of CA-US HIV RNA, the rapid decrease observed in the data may be a result of the loss of US HIV RNA by degradation and by splicing.

### Delayed Activation Model

Vorinostat treatment not only induces rapid changes in host gene expression but also induces changes after treatment cessation [10]. It is therefore plausible that the late increase in HIV transcription after vorinostat treatment is due to a longer-term impact on host gene transcription. To test this hypothesis, we extended the direct activation model to include a ‘transiently activated’ state (*L*_*T*_) and a ‘waiting’ state (*L*_*W*_), and denote this as the ‘delayed activation’ model. We assume that upon vorinostat treatment latently infected cells first get activated transiently, i.e., enter the transient activation state, *L*_*T*_, where CA-US HIV RNAs are produced for a short period of time. We assume the cells then enter a waiting state, *L*_*W*_, in which there is no CA-US HIV RNA production before becoming sustainably activated cells, *L*_*A*_ (Fig 1B). The waiting state reflects the time needed for the transcriptional programs to produce sufficiently high levels of host factors necessary for transcriptional activation such that the cellular environment becomes favorable for sustained HIV transcription. We assume that the cells in the transiently activated state and in the waiting state die at the same rate as in the latent state (*L*) as transient activation is not likely to be strong enough to produce the shock needed for kill. The ordinary differential equations (ODEs) describing this model are given in the Methods.

Next, we tested whether the above hypotheses explain both the short-term and the long-term dynamics of CA-US HIV RNAs by fitting the delayed activation model to the full data set (see S3 Table for best-fit parameter values), and found that the delayed activation model describes the data much better than the direct activation model in a majority of participants (compare S3 and S4 Figs with S1 and S2 Figs, respectively). It successfully describes the initial pattern of CA-US HIV RNA change following initiation of vorinostat in most individuals as well as the dynamics of CA-US HIV RNA in 6 of the 14 individuals where the level of CA-US HIV RNA increased after cessation of vorinostat. For the other 8 patients, the delayed activation model does not predict the magnitude of the late increase in CA-US HIV RNA level at some time points (S4 Fig). We speculate that the discrepancy may arise from the assumption of an exponentially distributed residence time for latently infected cells in the waiting state before becoming sustainably activated (an assumption implicitly assumed in the ODE system). This assumption is valid when a single event is needed for the transition to sustained activation. However, it is likely that multiple events must occur before the transition to sustained activation, such as upregulation of several host factors, HIV RNA splicing and expression of tat and other regulatory proteins including rev.

### Multistage Delayed Activation Model

We, thus, further modified our model to assume that cells in the waiting state have to go through several stages before becoming sustainably activated as in previous work describing the multiple events needed to drive an initially infected cell into viral production [48]. We denote this model the ‘multistage delayed activation’ model and the equations describing this model are given in the Methods. See Table 2 for a summary of the assumptions made with regard to the impact of vorinostat on latently infected cells in the three different models.

**Table 2:** Comparison of assumptions made in the direct activation, the delayed activation, and the multistage delayed activation model.

Direct activation model	Delayed activation model	Multistage delayed activation model
• Vorinostat is assumed to activate HIV transcription in latently infected cells differently in the three models. Cells that are transcriptionally induced by vorinostat are assumed to be in a different state than cells that are naturally activated.		
• Upon vorinostat treatment, latently infected cells become transcriptionally activated and express CA-US HIV RNA directly.	• Upon vorinostat treatment, latently infected cells are first activated transiently, where CA-US HIV RNAs are produced for a short period of time.	
	• After transient activation, latently infected cells enter a waiting state in which there is no CA-US HIV RNA production before becoming sustainably activated cells.	• After transient activation, latently infected cells go through several (n) waiting stages in which there is no CA-US HIV RNA production before becoming sustainably activated.

**Fig 3:**
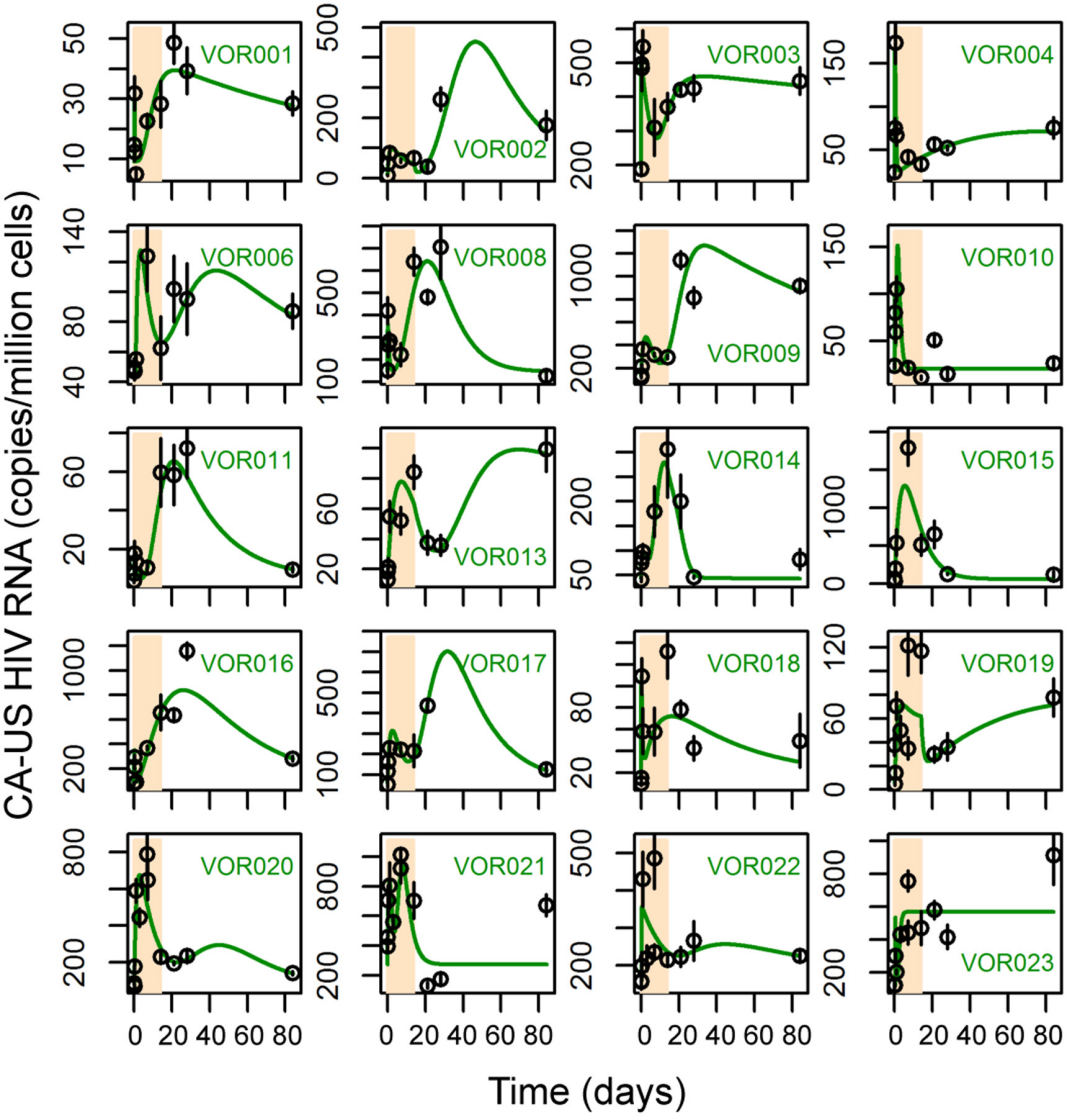
The multistage delayed activation model describes the clinical data well in a majority of the participants. Each panel shows the simulation trajectories using best-fit parameters of the multistage delayed activation model (green lines) and the levels of CA-US RNA measured in the clinical trial. The period of vorinostat treatment is shaded in bisque.

In this model, the *L*_*W*_ state is divided into *n* identical sub-states, i.e. *L*_W,1_, *L*_W,2_, … *L*_W,n_. The transition rate from one sub-state to the next is set to *nk*_*w*_ such that the average residence time in the overall waiting state is 1/*k*_*w*_. This model is equivalent to one in which we assume the transition out of the waiting state is stochastic with the delay described by a gamma probability distribution [49]. We let *n* change from 1 to 10, and fitted these 10 model variants to the clinical data from all 20 participants (S5 Fig). The fitting results show that this multistage delayed activation model describes the patterns of increases of CA-US HIV RNA after cessation of vorinostat as well as the initial peak following initiation of vorinostat (Fig 3 and S6 Fig; see S4 Table for best-fit parameter values).

We further performed model selection using the corrected Akaike information criterion (AICc) (see Methods). The direct activation model significantly underperformed compared to the delayed activation model and the multistage delayed activation model in 19 out of the 20 participants. The multistage delayed activation model was significantly better than the delayed activation model in 12 participants (Table 3). These results support the hypothesis that the immediate impact of vorinostat treatment is to activate HIV transcription for a short period of time (1–3 days), possibly due to the limited availability of many host cellular factors and that sustained activation may take longer to attain and a number of events (possibly in host cell transcriptional regulation) must occur before sustained HIV transcription becomes possible. As the number of events required varied among the participants, cells in different individuals may be in different states of latency. Also, host gene expression patterns, which can differ among individuals, may play a role in determining the length of the delay. Analyzing the effect of changing the number of waiting stages on the model fit to the data using AICc shows that for 17 out of the 20 participants, using a model with more than 7 stages would be a good choice in general (S5 Fig).

**Table 3:** The average residual sum of squares per data point (RSS/n) and relative AICc (ΔAICc) scores for the direct activation model, the delayed activation model and the multistage delayed activation model fit to the data in 20 patients. ΔAICc scores are calculated as the difference between the AICc score of a model and the AICc score of the best model in each patient, respectively. Thus ΔAICc = 0 indicates the best model. The total ΔAICc score is calculated as sum of ΔAICc scores in all 20 patients, where the lowest total ΔAICc indicates the best overall model.

Patient	RSS/n			ΔAICc		
	Direct activation model	Delayed activation model	Multistage delayed activation model	Direct activation model	Delayed activation model	Multistage delayed activation model
VOR001	0.21	0.15	0.11	13.5	8.1	0.0
VOR002	0.32	0.17	0.11	28.4	13.2	0.0
VOR003	0.06	0.04	0.03	15.1	4.3	0.0
VOR004	0.12	0.06	0.06	16.4	0.0	0.5
VOR006	0.10	0.09	0.07	1.7	5.4	0.0
VOR008	0.16	0.10	0.07	18.7	9.6	0.0
VOR009	0.22	0.04	0.02	70.9	19.7	0.0
VOR010	0.18	0.15	0.15	0.6	0.0	3.0
VOR011	0.25	0.18	0.12	18.3	11.0	0.0
VOR013	0.25	0.09	0.08	31.6	0.0	0.0
VOR014	0.16	0.15	0.13	0.0	3.1	0.7
VOR015	0.15	0.16	0.15	0.0	6.3	8.3
VOR016	0.17	0.06	0.03	53.0	23.4	0.0
VOR017	0.19	0.11	0.09	15.6	4.3	0.0
VOR018	0.41	0.17	0.18	26.8	0.0	5.2
VOR019	0.46	0.31	0.31	11.7	0.0	2.8
VOR020	0.06	0.07	0.05	4.6	14.9	0.0
VOR021	0.16	0.14	0.13	0.9	1.2	0.0
VOR022	0.08	0.06	0.05	7.1	3.1	0.0
VOR023	0.11	0.10	0.06	15.4	19.2	0.0
**Total ΔAICc**				**350.0**	**147.0**	**20.6**

### Quantitative Impact of Vorinostat Treatment on Latently Infected Cells

**Fig 4:**
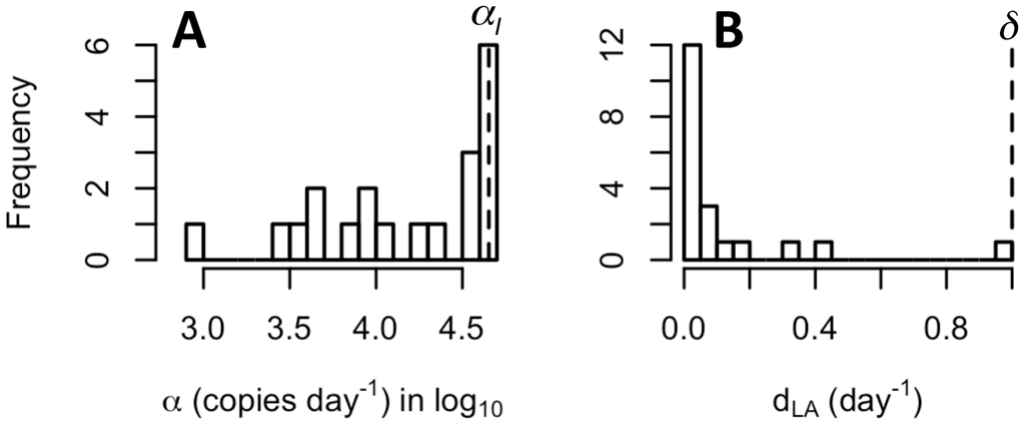
Distributions of best-fit values for the production rate of CA-US HIV RNA and the loss rate of sustainably activated cells in the 20 study participants. **(A)** The estimated production rates of CA-US HIV RNA, *α*, (in Log_10_) in transcriptionally activated latent cells. Dashed line shows the estimated production rate of CA-US RNA in productively infected cells, *α*_*I*_ = 4x10^4^ molecules day^-1^ [28] (see Methods). **(B)** The estimated loss rates of sustainably activated cells (*L*_*A*_), *d*_*LA*_. Dashed line shows the death rate of productively infected cells, *δ* = 1.0 day^-1^ [27].

We next examined the best-fit parameter values of the multistage delayed activation model to assess the impact of vorinostat on latently infected cells *in vivo*. First, we find that the estimated values of *α*, the rate of CA-US HIV RNA production induced by vorinostat in latently infected cells, varies over a wide range (over 1.5 logs) among the 20 patients, suggesting the response to vorinostat is very heterogeneous across participants. In a majority of patients, the estimated values of *α* are smaller than the production rate of CA-US HIV RNA in productively infected cells, *α*_*I*_ (Fig 4A; see Methods for calculation of *α*_*I*_). We then examined the estimated loss rate of transcriptionally activated latently infected cells, *d*_*LA*_, and found that in 12 participants the estimated loss rates are extremely low, close to the death rate of latently infected cells, *d*_*L*_ (Fig 4B). Although the estimated loss rates are higher than *d*_*L*_ in other patients, we were not able to distinguish whether the loss is through shutdown of HIV transcription or through cell death. Nonetheless, the low estimates of the loss rate in most participants suggest that vorinostat treatment does not induce killing of transcriptionally activated latent cells *in vivo* in a majority of individuals, and thus, according to this model, the reductions in reservoir size were minimal or absent in most participants.

We further tested the robustness of the parameter estimates to variations in our assumptions. First, we varied the values of two fixed parameter values that describe the intracellular dynamics of CA-US HIV RNAs, i.e. the rate of US HIV RNA export from the cell in the form of virions, *ρ*, which we initially assumed to be 0, and the combined rate of RNA splicing and degradation, *μ*. We find the parameter estimates are robust to changes in the value of *ρ* (S7 Fig), and that the estimated production rate of CA-US HIV RNAs, *α*, decreases approximately linearly with decreases in *μ* (S8 Fig). Thus, if the rate of CA-US HIV RNA loss is lower in cells activated by vorinostat than we have estimated, the estimated production rate of CA-US HIV RNA would also be lower. Last, we tested the robustness of our results to the assumption in the Rong *et al*. (15) model about how the latently infected cell population is maintained by employing a different model based on the work of Kim and Perelson [50] in which the latent population is maintained by homeostatic proliferation rather than asymmetric division (see Methods). We found the model fits to the CA-US HIV RNA data and the estimates of *α* and *d*_*L*_ are largely unaffected (S9 Fig).

## Discussion

We have constructed mathematical models to describe the dynamics of CA-US HIV RNA in HIV-infected individuals on ART who received multiple doses of the HDAC inhibitor vorinostat. By fitting these models to a clinical dataset, we have assessed the dynamic response of latently infected cells to vorinostat and estimated the quantitative impact of vorinostat on the latently infected cell population.

Model analyses show that the multistage delayed activation model, can describe both the short-term and the long-term patterns of change in CA-US HIV RNA induced by vorinostat in most individuals. This model assumes that in response to vorinostat treatment, HIV transcription in latently infected cells is induced transiently. Afterwards, the cells rather than returning to their original latent state go through several waiting stages where CA-US HIV RNAs are not produced but host gene expression patterns may change before becoming sustainably induced. The sustained induction of HIV transcription may even occur after vorinostat treatment is stopped. The induction of HIV gene expression depends on the availability of the HIV Tat protein as well as many host factors [34–36,42–44]. In latently infected cells, the number of Tat proteins [34] and the host factors necessary for inducing HIV transcription, such as P-TEFb, are likely to be at low levels [42–44], and at the same time, the presence of inhibitory molecules, such as Murr1, human schlafen 11 and tetherin, prevent transcriptional activation [45–47] before and at the early stage of response to vorinostat treatment. A recent proteomics and transcriptomics study showed that after 24 h of vorinostat treatment of primary CD4+ T cells the expression of a large number of host genes and proteins as well as genes and proteins previously reported to be involved in HIV transcription was modulated, with some effects appearing to be stimulatory and others inhibitory for HIV reactivation [51]. Therefore, it is likely that the immediate impact of vorinostat on histone acetylation and host gene transcription lead only to a transient induction of HIV RNA transcription and sustained HIV transcription may depend on the longer-term impact of vorinostat on host gene transcription [10]. This delay in sustained transcriptional induction may explain the later increase in the level of CA-US HIV RNA after cessation of vorinostat seen in this study, and the observed refractory periods in response to multiple doses of vorinostat in another study [52]. Note that the effect of vorinostat on host genes may also include the generation of read through transcripts containing HIV RNA [53], but a recent report suggests such transcripts are a minor fraction of total gag RNA [54].

Analyzing the model, we found that the number of stages latently infected cells goes through the waiting state and the total waiting period before sustained induced transcription varied among individuals. This suggests that latently infected cells in different individuals may be in different states, possibly due to variations in Tat protein copy number, host gene expression or alternatively different degrees of chromatin silencing or configuration potentially dependent on the sites of HIV integration. This, in turn, would cause responses to vorinostat to be heterogeneous. Interestingly, the maximal fold increase of CA-US HIV RNA was strongly correlated with the basal level of CA-US HIV RNA before vorinostat treatment [10]. Thus, it is plausible that the basal level of CA-US HIV RNA serves an indicator of the status of latency in a patient and the ease of induction of transcription using LRAs, suggesting that future treatment strategies may be able to be tailored to individual patients.

We further assessed the impact of vorinostat on the rate of loss of cells in the sustained activated state. This estimated loss rate, which serves as an upper bound on the death rate of activated cells (as cells could lose their activated state), is extremely low in most individuals, suggesting that vorinostat treatment does not induce killing of transcriptionally activated cells in most participants. This is in agreement with several previous *in vitro* and *ex vivo* studies showing vorinostat activates HIV transcription in only a subset of cells and that this level of HIV transcription and protein expression does not lead to cell death [21,52,53,55,56]. Interestingly, a recent *in vitro* study showed that vorinostat treatment only has significant impact on HIV transcriptional activation, with the impact on translation being minimal, suggesting that HIV proteins may not be produced sufficiently to lead to virion production or to induce viral cytopathic or cytotoxic T cell mediated cell death [20]. Thus, new treatment strategies aiming at both transcriptional and translational activation of HIV may be needed to induce efficient killing of latently infected cells.

In the model, we have assumed that in each participant, latently infected cells are a homogeneous population and respond to vorinostat by going through two activation steps. However, because of limited data sampling there could have been additional transient activation steps that we were unable to detect. In addition, the latent state of individual infected cells *in vivo* may differ [57] and it is possible that within an individual, some cells go through a different number of activation steps or have different waiting periods before becoming sustainably activated. This may give rise to the minor discrepancies between the data and the model seen in some participants (VOR010, VOR018, VOR019, VOR021, VOR023 in Fig 3). Although a model that accounts for different responses of latently infected cell subpopulations or has additional activation steps might explain the data, such a model would have more unknown parameters than our current model with only a marginal improvement in model fit. Nonetheless, the possibility that there exist different cell populations in individual patients in terms of their response to vorinostat or additional activation steps cannot be excluded. Further experiments examining the dynamics of host factors/enzymes that are responsible for transcriptional activation and inhibition under LRA treatment could validate our model, improve our understanding of the impact of vorinostat, and ultimately aid the design of treatment strategies to eradicate the latent reservoir.

To conclude, our results suggest that vorinostat induces both immediate transient induction and delayed sustained induction of HIV transcription. Similar dynamic patterns of CA-US HIV RNA were also observed in clinical trials of the LRAs panobinostat and romidepsin [58,59], suggesting LRAs may induce both transient and delayed transcription activation in latently infected cells in general. Therefore, designing clinical trials with frequent longitudinal sampling during both treatment and the follow-up period would help quantify the impact of LRAs. To our knowledge, our work represents the first mathematical model to assess the impact of a LRA on the dynamics of CA-US HIV RNA *in vivo*. Our model can be easily adapted to study other LRAs as well as combinations of these agents once data are available. In addition, our model or variants of it could be used to assess the efficacy of different candidate treatments, such as those using anti-HIV monoclonal antibodies combined with LRAs, and ultimately suggest optimal drug combinations to eliminate latently infected cells in HIV-infected individuals.

## Methods

### Clinical Data

As described by Elliot *et al*. [10], 20 chronically HIV-infected adults receiving at least three antiretroviral agents, having plasma HIV RNA < 50 copies per mL for at least three years (excluding single viral ‘blips’), a CD4+ T-cell count > 500 cells/μL and documented subtype B HIV-1 infection were recruited into a vorinostat trial (ClinicalTrials.gov, NCT01365065). Participants received vorinostat 400 mg orally once daily for 14 days. Levels of CA-US HIV RNA were measured in peripheral blood mononuclear cells at 0, 2, 8 and 24 hours, and on days 7, 14, 21, 28 and 84 (as well as on day 3 for participants VOR019-023). For each blood sample, four replicate q-PCR runs were performed to measure the levels of CA-US HIV RNA. The total number of data points in each patient ranges from 35 to 44.

### The Direct Activation Model

The ordinary differential equations (ODEs) describing the model are:

$$\begin{array}{l} \frac{dT}{dt}=s-d_{T}T-(1-\varepsilon_{RT})\beta V_{I}T\\ \frac{dI}{dt}=(1-\varepsilon_{RT})(1-f)\beta V_{I}T-\delta I^{1.44}+2(1-p_{L})aL\\ \frac{dL}{dt}=(1-\varepsilon_{RT})f\beta V_{I}T-d_{L}L-aL+2p_{L}aL-\upsilon L\\ \frac{dL_{A}}{dt}=\upsilon L-d_{LA}L_{A}\\ \frac{dR}{dt}=\alpha -\mu R-\rho R\\ \frac{dV_{I}}{dt}=(1-\varepsilon_{PI})\left[p_{V}I+\rho RL_{A}\right]-cV_{I}\\ \frac{dV_{NI}}{dt}=\varepsilon_{PI}\left[p_{V}I+\rho RL_{A}\right]-cV_{NI}\\ US=RL_{A}+US_{0} \end{array}$$

In this model, target cells, *T*, are produced at a constant rate, *s*, and die at per capita rate, *d*_*T*_. In the absence of cART, they are infected at per capita rate, *βV*_*I*_, where *β* is a rate constant and *V*_*I*_ is the concentration of infectious virus. The effect of reverse transcriptase inhibitors (RTI) is to multiply the rate of infection by the factor 1-*ε*_*RT*_, where *ε*_*RT*_ is the effectiveness of the RTI with 0 ≤ *ε*_*RT*_ *≤* 1. Under protease inhibitor treatment, a fraction, *ε*_*PI*_, of produced viruses are non-infectious (*V*_*NI*_). Upon infection with viruses, a fraction, *f*, of infected cells becomes latently infected, and the remaining fraction, 1-*f*, becomes productively infected. Productively infected cells, *I*, die at per capita rate, *δI* ^1.44^, where the power 1.44 models the effects of both viral cytopathicity and cell-mediated immune responses [60,61].

We assume that latently infected cells, *L*, when activated by antigen at rate *a*, undergo an asymmetric division in which a daughter cell remains in latency with probability, *p*_*L*_, and becomes productively infected and produces virus with probability, 1-*p*_*L*_ [15]. Latently infected cells die at per capita rate, *d*_*L*_. We assume that vorinostat causes latently infected cells to move into a transcriptionally activated state, *L*_*A*_, at rate *ν*. The transcriptionally activated cells are lost at rate, *d*_*LA*_. We also assume there is a pharmacological delay, *t*_*0*_, in the effect of vorinostat upon treatment initiation such that the rate of activation remains 0 when *t*<*t*_0_, and it becomes *ν* when *t*_0_≤*t*≤14 days. After treatment is terminated, we assume the effectiveness declines exponentially as *ν*e^*-w(t-14)*^ for *t*>14 days, where *w* is the rate at which vorinostat is cleared from the system. We also model the population average of the amount of CA-US HIV RNA (*R*) within transcriptionally activated cells. We assume that CA-US HIV RNA is produced at a constant rate, *α*, once vorinostat becomes effective, i.e. when *t*≥ *t*_0_. CA-US HIV RNA is encapsidated and exported as virions at per capita rate, *ρ*, and lost by degradation and splicing at per capita rate, *μ*. Here, we assume that the transcriptionally activated cells do not produce mature viral particles, i.e. *ρ* = 0, because recent work shows that the production of virus from cells treated with vorinostat is minimal [20,53]. As other LRAs may induce viral production, we leave *ρ* in the model. Viruses are produced from productively infected cells at rate *p*_*v*_ and from transcriptionally activated cells at rate *ρR* (note that *ρ* is set to 0 for vorinostat). Viruses are cleared at per capita rate *c*. The amount of CA-US HIV RNAs, *US*, is calculated as the sum of the basal level of CA-US HIV RNA, *US*_0_, and the average number of US HIV RNAs per activated cell, *R*, multiplied by the number of activated cells, *L*_*A*_.

### The Delayed Activation Model

In the delayed activation model, the equations describing the transient activation state, *L*_*T*_, the waiting state, *L*_*W*_, the transcriptionally activated state, *L*_*A*_, and the total number of CA-US HIV RNAs, *US*, are

$$\begin{array}{l} \frac{dL_{T}}{dt}=\upsilon L-d_{L}L_{T}-k_{T}L_{T}\\ \frac{dL_{W}}{dt}=k_{T}L_{T}-d_{L}L_{W}-k_{W}L_{W}\\ \frac{dL_{A}}{dt}=k_{W}L_{W}-d_{LA}L_{A}\\ US=R(L_{T}+L_{A})+US_{0} \end{array}$$

where *k*_*T*_ and *k*_*w*_ are transition rate constants. The ODEs describing other variables are kept the same as in the direct activation model.

### The Multistage Delayed Activation Model

The ODEs describing the multistage waiting states are:

$$\begin{array}{l} \frac{dL_{W,1}}{dt}=k_{T}L_{T}-d_{L}L_{W,1}-nk_{W}L_{W,1}\\ \frac{dL_{W,i}}{dt}=nk_{W}L_{W,i-1}-d_{L}L_{W,i}-nk_{W}L_{W,i}\;i=2,3,\ldots \ldots ,n\\ \frac{dL_{A}}{dt}=nk_{W}L_{W,n}-d_{LA}L_{A} \end{array}$$

where the waiting state is divided into *n* identical sub-states, i.e. *L*_W,1_, *L*_W,2_, … *L*_W,n_. The transition rate from one sub-state to the next is set to *nk*_*w*_, so that the average residence time in the waiting state, 1/*k*_*w*_, is the same as in the delayed activation model. The ODEs describing other variables are the same as in the delayed activation model.

### A Model Assuming Constant Proliferation of Latently Infected Cells

To test the assumption about how the population of latently infected cells is maintained, we modified the multi-stage delayed activation such that the latently infected cells proliferate at a constant rate as in Kim and Perelson [50] instead of asymmetric division in the main text. The ordinary differential equations (ODEs) describing the productively infected population and latently infected population are shown below and other terms in the model are kept the same as in the main text.

$$\begin{array}{l} \frac{dI}{dt}=(1-\varepsilon_{RT})(1-f)\beta V_{I}T-\delta I^{1.44}+aL\\ \frac{dL}{dt}=(1-\varepsilon_{RT})f\beta V_{I}T-d_{L}L-aL+rL-\upsilon L \end{array}$$

In this model, the latently infected cells becomes activated at per capita rate *a* and proliferate at per capita rate *r*. We set *a* = 0.0088 day^-1^ according to [62] and *r* = 0.0183 day^-1^, such that the half-life of the latent reservoir is 44 months as estimated before [63].

### Parameter Values

The fixed parameter values are based on prior work to yield a baseline state with a plasma viral load of 5 copies/mL and a latently infected cell population of ~2 cells/million cells (Table 1). The production and death rates of uninfected CD4^+^ T cells were chosen to yield a baseline CD4 count similar to the average level in patients in Elliot *et al*. [10]. Changes in the values of the fixed parameters that govern the dynamics of target cells and productively infected cells do not impact the estimation of the fitted parameters (S10 Fig). This is because cART is so effective that the contribution of new infections to the latent reservoir is negligible over the time period we study here. We also tested the robustness of the parameter estimates against changes in the fixed parameters that govern intracellular HIV transactivation in latently infected cells and to the structure of the model with regard to how latently infected cells proliferate (S7–S9 Figs).

The CA-US HIV RNA production rate in a productively infected cell, *α*_*I*_, is calculated based on the derivations in Ref. [28]. The basal transcription rate in eukaryotic cells is estimated to be approximately 40 nucleotides/s [64]. The HIV genome has around 9500 nucleotides. Thus, the basal production rate of CA-US HIV RNA production can be estimated as 40/9500≈0.0047 s^-1^≈406 day^-1^. In the presence of Tat, the transcription is upregulated to 100 fold [65]. Then, the production rate of CA-US HIV RNA in a productively infected cell, *α*_0_, can be calculated as approximately 40,000 transcripts day^-1^. This maybe a minimal estimate if productively infected cells live approximately one day while producing virus, as Chen *et al*. [32] have estimated that a productively infected cell produces between 40,000 and 55, 000 virions over its lifespan.

### Model Fitting and Model Selection

To fit the direct activation model to the data, we varied 5 unknown model parameters: the production rate of CA-US HIV RNA in a transcriptionally activated cell (*α*), the death rate of transcriptionally activated cells (*d*_*LA*_), the rate of transcriptional activation (*ν*), the initial delay of vorinostat effectiveness (*t*_0_) and the baseline US HIV RNA level (*US*_0_). To fit the delayed activation model and the multistage delayed activation model, we allow 2 additional parameters to be estimated (together with the 5 parameters in the direct-activation model): the transition rate, *k*_*T*_, from the transiently activated state to the waiting state, and the transition rate, *k*_*w*_, from one sub-state of the waiting state to the next.

To estimate the parameter values in each model, we first calculate the residual sum of squares (RSS) between model predicted log CA-US RNA level and log transformed data, and then minimize the RSS using the Nelder-Mead algorithm [66]. 1,000 individual fits were performed for each model starting from parameter values randomly sampled within biologically plausible ranges. The parameter values with the smallest RSS among the 1,000 fits are taken as the best-fit parameter values for each model.

We perform model selection using the corrected Akaike information criterion (AICc) to account for the low number of data points (ranges from 35 to 44) for each patient [67]. The AICc score is calculated as

$$AICc=n\text{log}(\frac{RSS}{n})+2K\frac{n}{n-K-1}$$

where *n* is the number of data points and *K* is the number of fitted parameters. When comparing models, the model with the lowest score is the best model, although small difference in AICc scores, e.g. ≤ 2, is not significant [67].

## Supporting Information

S1 Fig Fitting results of the direct activation model to the entire 84-day clinical data (only the dynamics during the first 8 days of treatment are shown). Each panel shows the fitting result for a patient. Red lines are model simulations using best-fit parameter values. The black circles and vertical black lines are the mean and standard deviation of four replicate measurements at different time points.
(PDF)

S2 Fig Fitting results of the direct activation model to the clinical data using the full data set. The same simulation results as in S1 Fig; however, the dynamics during the entire 84 days of study period are shown. Each panel shows the fitting result for a patient. Red lines are model simulations using best-fit parameter values. The black circles and vertical black lines are the mean and standard deviation of four replicate measurements at different time points.
(PDF)

S3 Fig The delayed activation model describes the pattern of CA-US HIV RNAs during the initial treatment period well. Plots show the best-fit results of the delayed activation model to the entire 84-day clinical data. Blue lines are model simulations using best-fit parameter values. The black circles and vertical black lines are the mean and standard deviation of four replicate measurements at different time points.
(PDF)

S4 Fig The delayed activation model describes the later increase of CA-US HIV RNA after treatment cessation well in general. Among the 14 patients where the level of CA-US RNA increases after treatment is stopped, this model describes the pattern well in 6 patients (VOR001, VOR003, VOR004, VOR011, VOR013 and VOR019). It does not describe the amplitude of the increase in 8 patients (VOR002, VOR008, VOR009, VOR016-VOR018, VOR021 and VOR023). Plots show the same simulation results as in S3 Fig; however, the entire 84 days of study period are shown. Each panel shows the simulation trajectories using best-fit parameters of the delayed activation model (blue lines), and the levels of CA-US RNA measured in clinical trials. The period of vorinostat treatment is shaded in bisque.
(PDF)

S5 Fig Fitting the multistage delayed activation model using a range of number of stages, *n* (*n* = 1–10). **(A)** The relative AICc scores for the 10 model variants with different values of *n* in all 20 patients. Each colored line represents the relative AICc scores of the 10 model variants in each patient. The dashed horizontal line shows the threshold ΔAICc = 2, above which the model variant performs significantly worse than the best model. **(B)** The distribution of the value of *n* in the best-fit of the multistage model to the data from all 20 patients. Note that, for most of the patients (17 out of 20), a model that assumes the number of waiting stages is greater than 7 would be a good model to describe the data in general.
(PDF)

S6 Fig The multistage delayed activation model describes the first 7-day data well in all patients. The same simulation results as shown in Fig 3 except that the x-axis is scaled to show the agreement between the model and for the first 7-day data.
(PDF)

S7 Fig Parameter estimation is robust to changes in the assumption of the virion export rate, *ρ*. Plots show the comparisons of parameter estimates of the rate of CA-US HIV RNA production, *α* in log_10_ (left panel) and the loss rate of sustainably activated cells, *d*_*LA*_ (right panel) between the multistage delayed activation model in the main text (‘without virion production’) with a model assuming that activated cells produces virions at the same rate as in the productively infected cells (‘virion production’). In each panel, a dot represents a pair of estimates in one patient, and the dashed lines show the line for y = x.
(PDF)

S8 Fig Estimations of the rate of CA-US HIV RNA production, *α*, change approximately linearly with changes in the assumed combined rate of HIV RNA splicing and degradation, *μ*, while estimations of the loss rate of sustainably activated cells, *d*_*LA*_ are robust to changes in *μ*. Plots show the comparisons of parameter estimates of *α* in log_10_ (left panel) and *d*_*LA*_ (right panel) between the multistage delayed activation model in the main text (‘baseline splicing’) with a model assuming that the combined rate of splicing and degradation is 10% of its original value. In each panel, a dot represents a pair of estimates in one patient. The dashed line in the left panel shows the line for y = x-1, and this correspond to 90% of reduction in the estimated value of *α* (on a log10 scale), while the dashed line in the right panel shows the line for y = x. Note that, since the dynamics of intracellular CA-US HIV RNA reaches equilibrium very quickly in the time scale we are considering in this study, the approximately linear relationship between *α* and *μ* can be easily seen from the ODE describing the dynamics of intracellular CA-US HIV RNA by deriving the steady state equation.
(PDF)

S9 Fig Parameter estimation is robust to changes in the assumption of how latently infected cells are maintained in the absence of vorinostat treatment. Plots show the comparisons of parameter estimates of the rate of CA-US HIV RNA production, *α* in Log_10_ (left panel) and the loss rate of sustainably activated cells, *d*_*LA*_ (right panel) between the multistage delayed activation model in the main text (‘asymmetric division’) with a model assuming constant proliferation of latently infected cells as in Kim & Perelson [50] (‘Constant proliferation’). In each panel, a dot represents a pair of estimates in one patient, and the dashed lines show the line for y = x.
(PDF)

S10 Fig Sensitivity analysis for changes in the values of fixed parameters governing the dynamics of target cells and infected cells, i.e. *s* (in panel A), *β* (in panel B), *δ* (in panel C) and *p*_*V*_ (in panel D). Simulation results using best-fit parameter values (lines) for Patient VOR001 are shown. Diamonds and ‘x’s show the simulation results using parameter values with a 2-fold increase or 2-fold decrease from the best-fit parameters, respectively.
(PDF)

S1 Table Best fit parameter values of the direct activation model to the full dataset in each patient. (PDF)

S2 Table Best fit parameter values of the direct activation model to the data from the first 7-day treatment in each patient. (PDF)

S3 Table Best fit parameter values of the delayed activation model to the full data set in each patient. (PDF)

S4 Table Best fit parameter values of the multistage delayed activation model to the full data set in each patient. (PDF)
