# Supplementary material for: Modeling the Effects of Vorinostat In Vivo Reveals both Transient and Delayed HIV Transcriptional Activation and Minimal Killing of Latently Infected Cells
Source: PLoS Pathog. 2015 Oct 23;11(10):e1005237. doi: 10.1371/journal.ppat.1005237 (PMC4619772; doi:10.1371/journal.ppat.1005237)
Supplement: S1 Fig — Each panel shows the fitting result for a patient. Red lines are model simulations using best-fit parameter values. The black circles and vertical black lines are the mean and standard deviation of four replicate measurements at different time points. (PDF) [file ppat.1005237.s001.pdf]

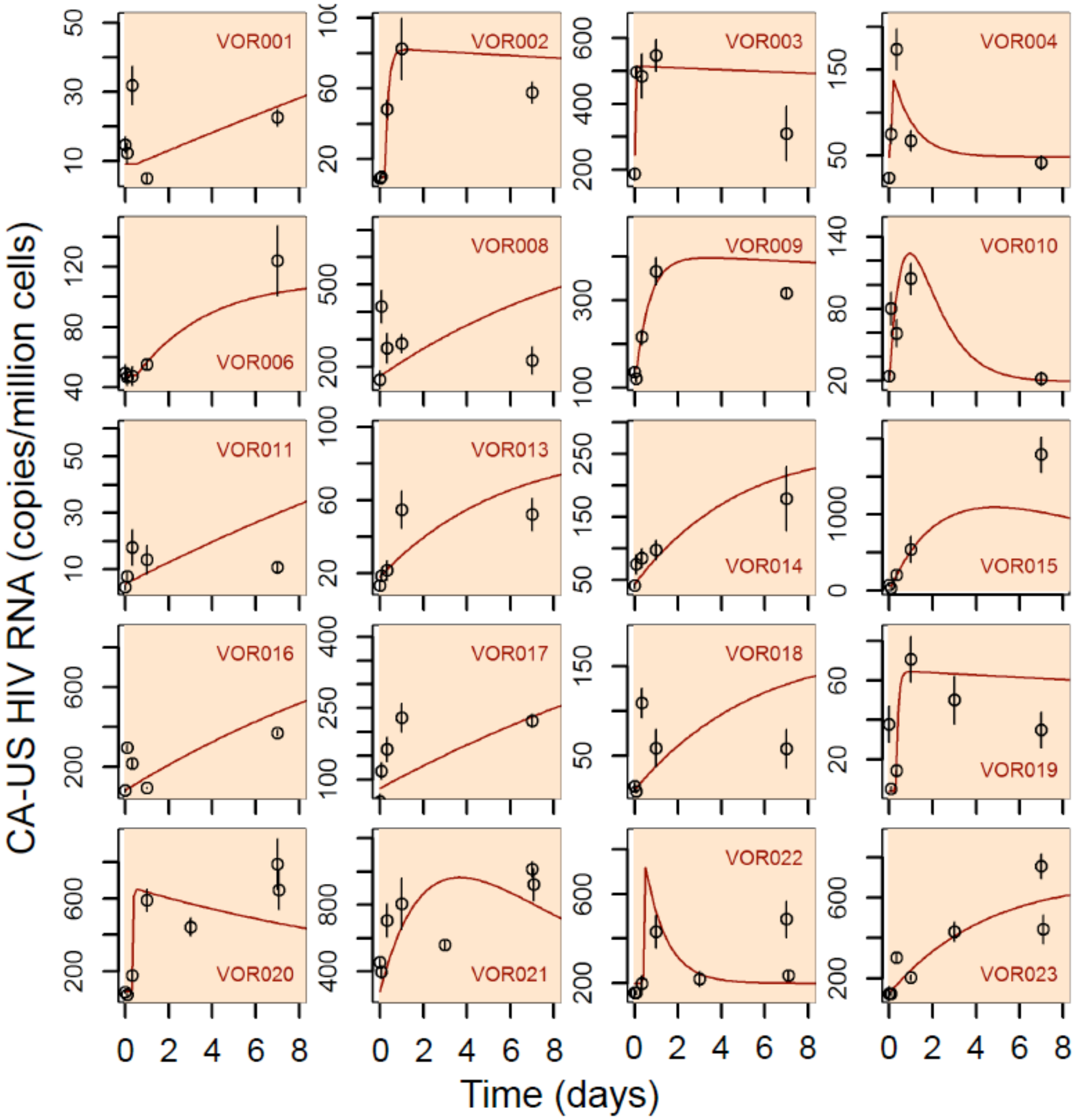

Figure S1. Fitting results of the direct activation model to the entire 84-day clinical data (only the dynamics during the first 8 days of treatment are shown). Each panel shows the fitting result for a patient. Red lines are model simulations using best-fit parameter values. The black circles and vertical black lines are the mean and standard deviation of four replicate measurements at different time points.
