# Supplementary material for: Modeling the Effects of Vorinostat In Vivo Reveals both Transient and Delayed HIV Transcriptional Activation and Minimal Killing of Latently Infected Cells
Source: PLoS Pathog. 2015 Oct 23;11(10):e1005237. doi: 10.1371/journal.ppat.1005237 (PMC4619772; doi:10.1371/journal.ppat.1005237)
Supplement: S3 Fig — Plots show the best-fit results of the delayed activation model to the entire 84-day clinical data. Blue lines are model simulations using best-fit parameter values. The black circles and vertical black lines are the mean and standard deviation of four replicate measurements at different time points. (PDF) [file ppat.1005237.s003.pdf]

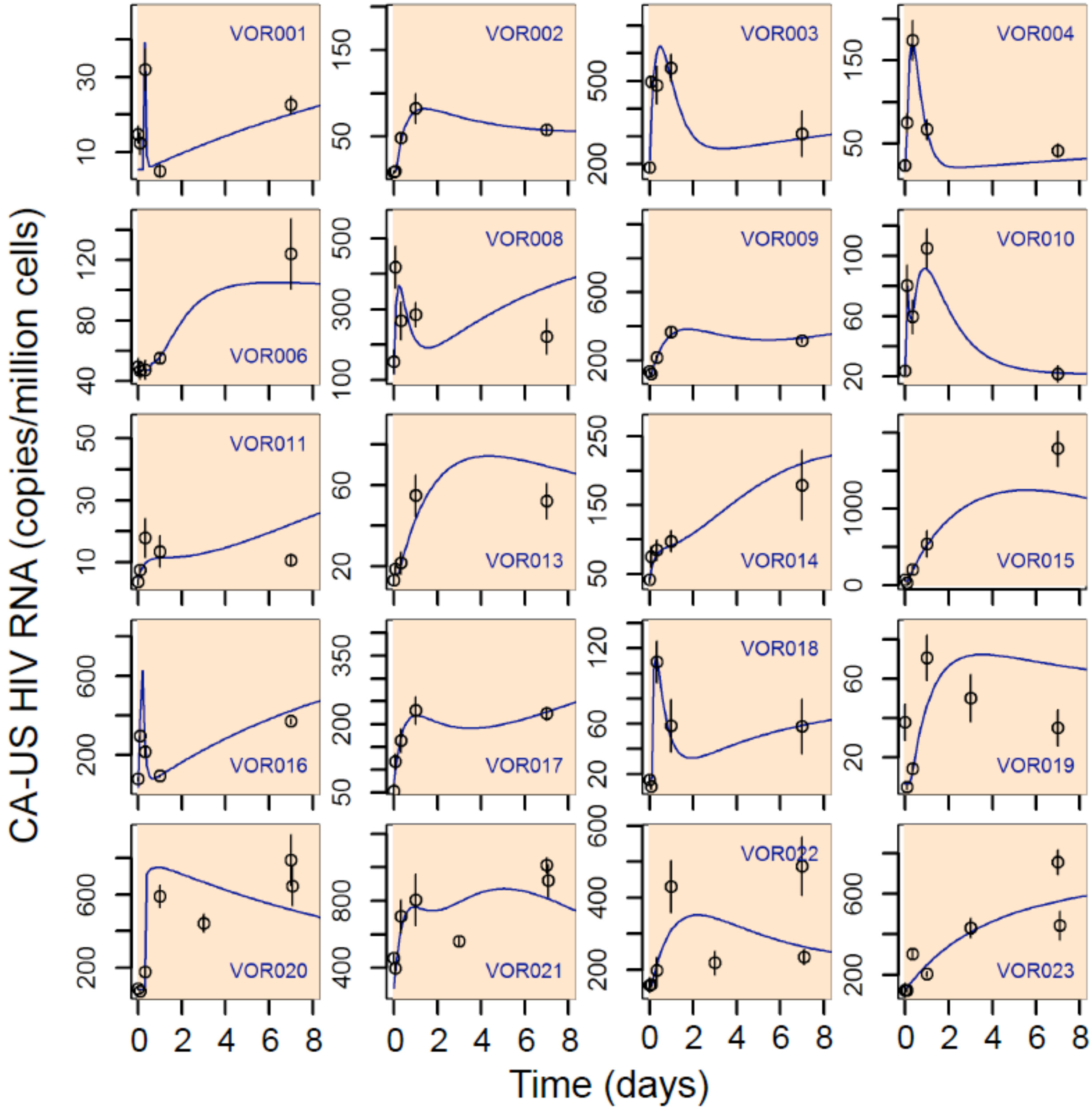

Figure S3. The delayed activation model describes the pattern of CA-US HIV RNAs during the initial treatment period well. Plots show the best-fit results of the delayed activation model to the entire 84-day clinical data. Blue lines are model simulations using best-fit parameter values. The black circles and vertical black lines are the mean and standard deviation of four replicate measurements at different time points.
