# Supplementary material for: Modeling the Effects of Vorinostat In Vivo Reveals both Transient and Delayed HIV Transcriptional Activation and Minimal Killing of Latently Infected Cells
Source: PLoS Pathog. 2015 Oct 23;11(10):e1005237. doi: 10.1371/journal.ppat.1005237 (PMC4619772; doi:10.1371/journal.ppat.1005237)
Supplement: S4 Fig — Among the 14 patients where the level of CA-US RNA increases after treatment is stopped, this model describes the pattern well in 6 patients (VOR001, VOR003, VOR004, VOR011, VOR013 and VOR019). It does not describe the amplitude of the increase in 8 patients (VOR002, VOR008, VOR009, VOR016-VOR018, VOR021 and VOR023). Plots show the same simulation results as in S3 Fig; however, the entire 84 days of study period are shown. Each panel shows the simulation trajectories using best-fit parameters of the delayed activation model (blue lines), and the levels of CA-US RNA measured in clinical trials. The period of vorinostat treatment is shaded in bisque. (PDF) [file ppat.1005237.s004.pdf]

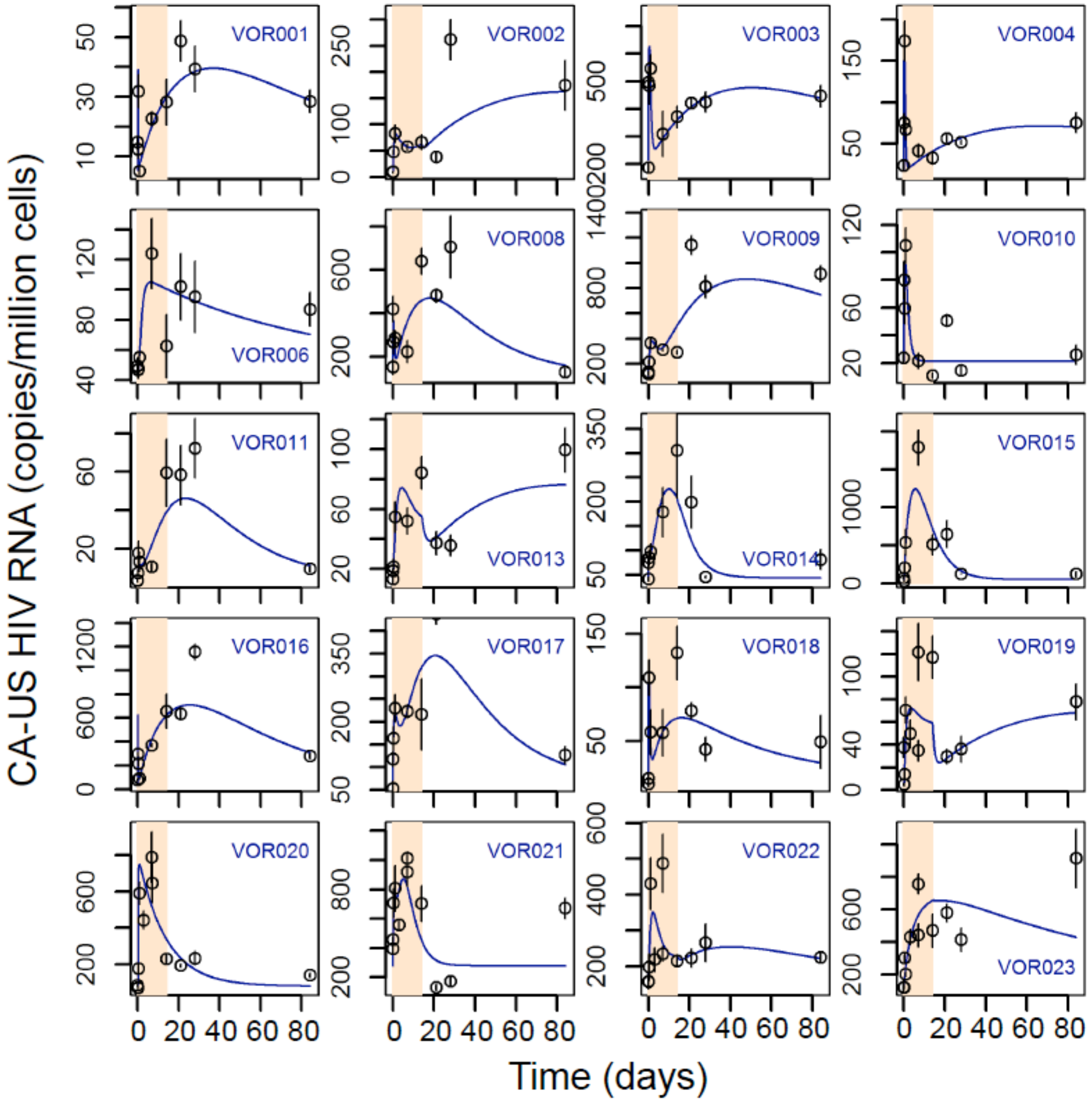

Figure S4. The delayed activation model describes the later increase of CA-US HIV RNA after treatment cessation well in general. Among the 14 patients where the level of CA-US RNA increases after treatment is stopped, this model describes the pattern well in 6 patients (VOR001, VOR003, VOR004, VOR011, VOR013 and VOR019). It does not describe the amplitude of the increase in 8 patients (VOR002, VOR008, VOR009, VOR016-VOR018, VOR021 and VOR023). Plots show the same simulation results as in Fig. S3; however, the entire 84 days of study period are shown. Each panel shows the simulation trajectories using best-fit parameters of the delayed activation model (blue lines), and the levels of CA-US RNA measured in clinical trials. The period of vorinostat treatment is shaded in bisque.
