# Supplementary material for: Modeling the Effects of Vorinostat In Vivo Reveals both Transient and Delayed HIV Transcriptional Activation and Minimal Killing of Latently Infected Cells
Source: PLoS Pathog. 2015 Oct 23;11(10):e1005237. doi: 10.1371/journal.ppat.1005237 (PMC4619772; doi:10.1371/journal.ppat.1005237)
Supplement: S5 Fig — (A) The relative AICc scores for the 10 model variants with different values of n in all 20 patients. Each colored line represents the relative AICc scores of the 10 model variants in each patient. The dashed horizontal line shows the threshold ΔAICc = 2, above which the model variant performs significantly worse than the best model. (B) The distribution of the value of n in the best-fit of the multistage model to the data from all 20 patients. Note that, for most of the patients (17 out of 20), a model that assumes the number of waiting stages is greater than 7 would be a good model to describe the data in general. (PDF) [file ppat.1005237.s005.pdf]

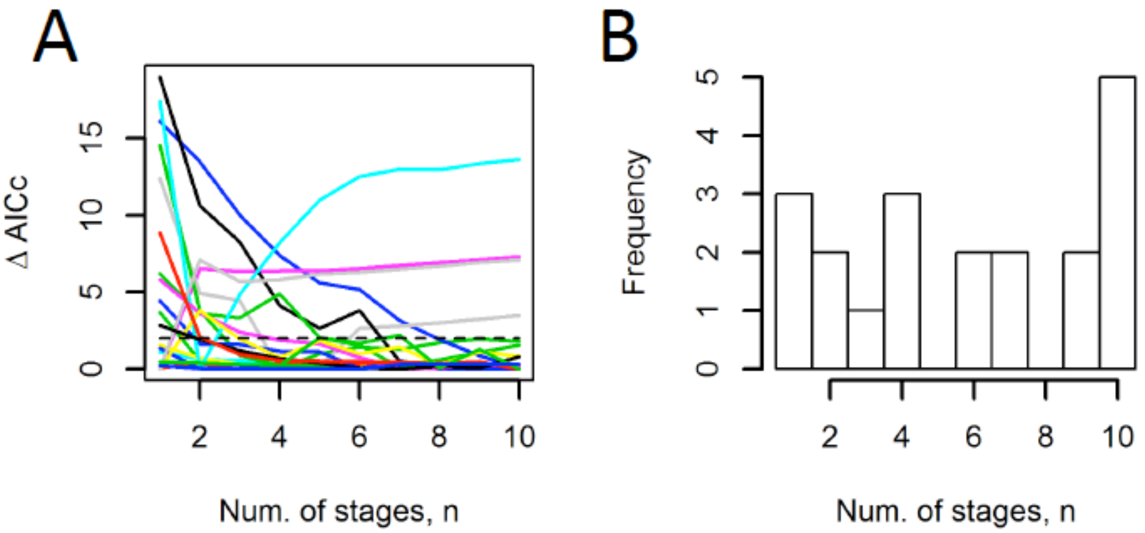

Figure S5. Fitting the multistage delayed activation model using a range of number of stages, *n* (*n*=1-10). (A) The relative AICc scores for the 10 model variants with different values of *n* in all 20 patients. Each colored line represents the relative AICc scores of the 10 model variants in each patient. The dashed horizontal line shows the threshold ΔAICc=2, above which the model variant performs significantly worse than the best model. **(B)** The distribution of the value of *n* in the best-fit of the multistage model to the data from all 20 patients. Note that, for most of the patients (17 out of 20), a model that assumes the number of waiting stages is greater than 7 would be a good model to describe the data in general.
