# Supplementary material for: Modeling the Effects of Vorinostat In Vivo Reveals both Transient and Delayed HIV Transcriptional Activation and Minimal Killing of Latently Infected Cells
Source: PLoS Pathog. 2015 Oct 23;11(10):e1005237. doi: 10.1371/journal.ppat.1005237 (PMC4619772; doi:10.1371/journal.ppat.1005237)
Supplement: S6 Fig — The same simulation results as shown in Fig 3 except that the x-axis is scaled to show the agreement between the model and for the first 7-day data. (PDF) [file ppat.1005237.s006.pdf]

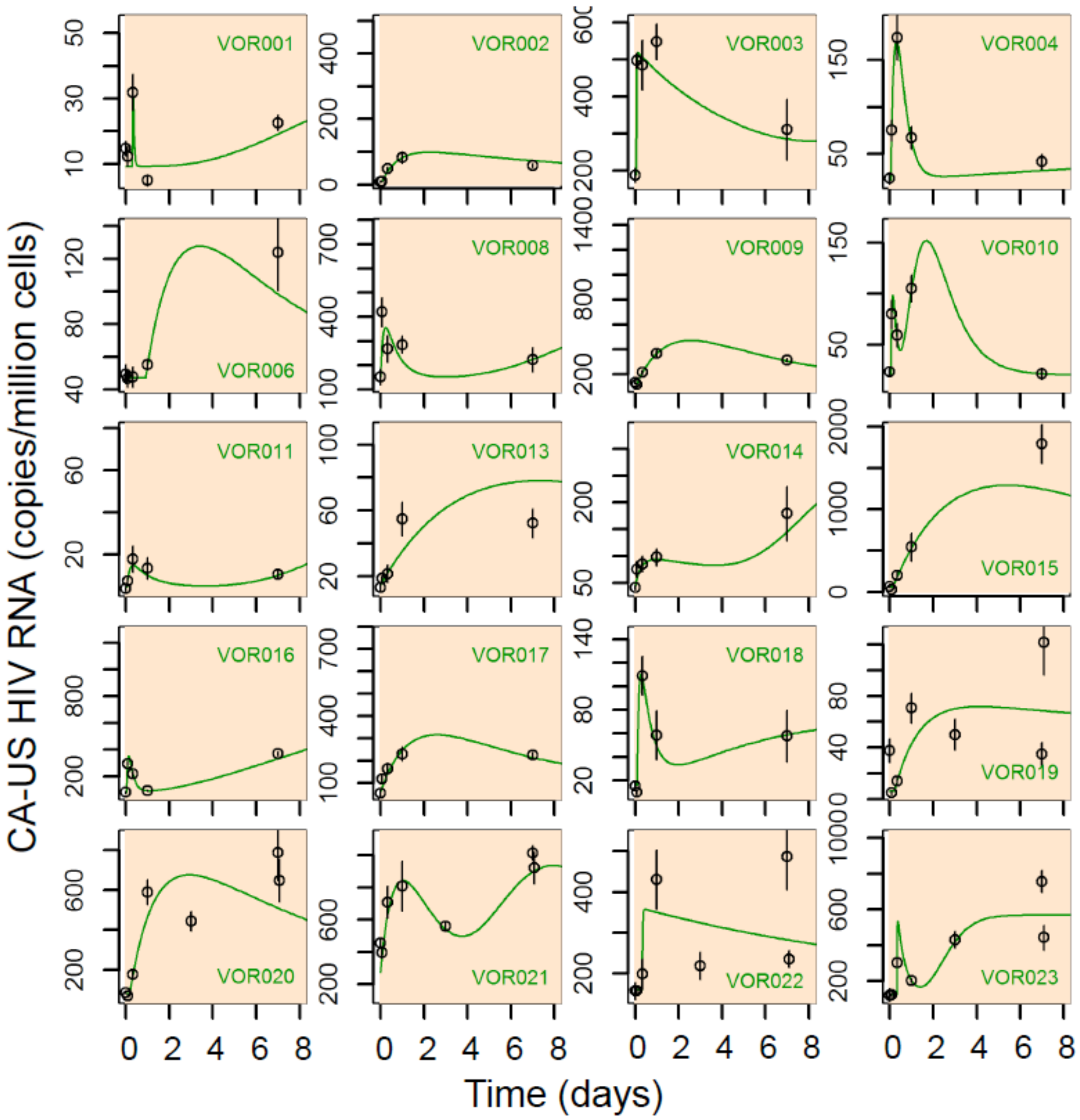

Figure S6. The multistage delayed activation model describes the first 7-day data well in all patients. The same simulation results as shown in Fig.3 except that the x-axis is scaled to show the agreement between the model and for the first 7-day data.
