# Supplementary material for: Modeling the Effects of Vorinostat In Vivo Reveals both Transient and Delayed HIV Transcriptional Activation and Minimal Killing of Latently Infected Cells
Source: PLoS Pathog. 2015 Oct 23;11(10):e1005237. doi: 10.1371/journal.ppat.1005237 (PMC4619772; doi:10.1371/journal.ppat.1005237)
Supplement: S7 Fig — Plots show the comparisons of parameter estimates of the rate of CA-US HIV RNA production, α in log10 (left panel) and the loss rate of sustainably activated cells, d LA (right panel) between the multistage delayed activation model in the main text (‘without virion production’) with a model assuming that activated cells produces virions at the same rate as in the productively infected cells (‘virion production’). In each panel, a dot represents a pair of estimates in one patient, and the dashed lines show the line for y = x. (PDF) [file ppat.1005237.s007.pdf]

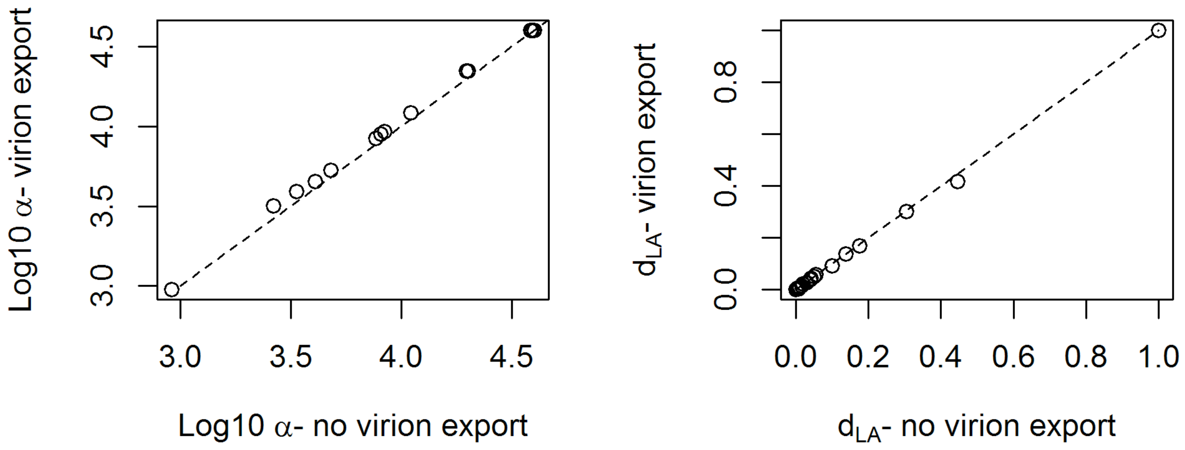

Figure S7. Parameter estimation is robust to changes in the assumption of the virion export rate, ρ. Plots show the comparisons of parameter estimates of the rate of CA-US HIV RNA production, α in Log_{10} (left panel) and the loss rate of sustainably activated cells, d_{LA} (right panel) between the multistage delayed activation model in the main text ('without virion production') with a model assuming that activated cells produces virions at the same rate as in the productively infected cells ('virion production'). In each panel, a dot represents a pair of estimates in one patient, and the dashed lines show the line for y=x.
