# Supplementary material for: Modeling the Effects of Vorinostat In Vivo Reveals both Transient and Delayed HIV Transcriptional Activation and Minimal Killing of Latently Infected Cells
Source: PLoS Pathog. 2015 Oct 23;11(10):e1005237. doi: 10.1371/journal.ppat.1005237 (PMC4619772; doi:10.1371/journal.ppat.1005237)
Supplement: S8 Fig — Plots show the comparisons of parameter estimates of α in log10 (left panel) and d LA (right panel) between the multistage delayed activation model in the main text (‘baseline splicing’) with a model assuming that the combined rate of splicing and degradation is 10% of its original value. In each panel, a dot represents a pair of estimates in one patient. The dashed line in the left panel shows the line for y = x-1, and this correspond to 90% of reduction in the estimated value of α (on a log10 scale), while the dashed line in the right panel shows the line for y = x. Note that, since the dynamics of intracellular CA-US HIV RNA reaches equilibrium very quickly in the time scale we are considering in this study, the approximately linear relationship between α and μ can be easily seen from the ODE describing the dynamics of intracellular CA-US HIV RNA by deriving the steady state equation. (PDF) [file ppat.1005237.s008.pdf]

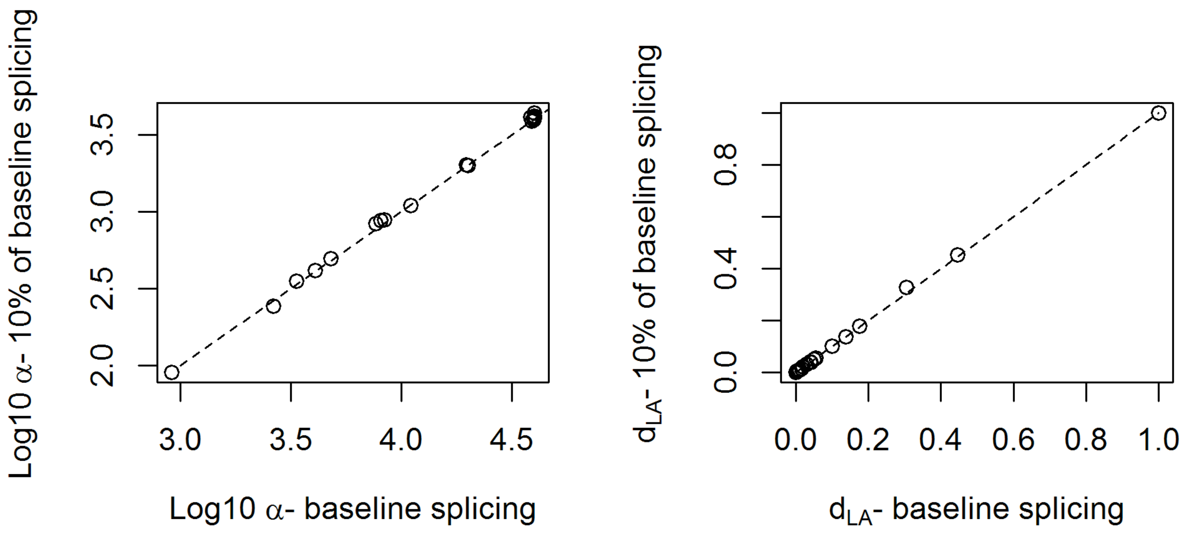

Figure S8. Estimations of the rate of CA-US HIV RNA production, α, change approximately linearly with changes in the assumed combined rate of HIV RNA splicing and degradation, μ, while estimations of the loss rate of sustainably activated cells, d_{LA} are robust to changes in μ. Plots show the comparisons of parameter estimates of α in Log_{10} (left panel) and d_{LA} (right panel) between the multistage delayed activation model in the main text ('baseline splicing') with a model assuming that the combined rate of splicing and degradation is 10% of its original value. In each panel, a dot represents a pair of estimates in one patient. The dashed line in the left panel shows the line for y=x-1, and this correspond to 90% of reduction in the estimated value of α (on a log10 scale), while the dashed line in the right panel shows the line for y=x. Note that, since the dynamics of intracellular CA-US HIV RNA reaches equilibrium very quickly in the time scale we are considering in this study, the approximately linear relationship between α and μ can be easily seen from the ODE describing the dynamics of intracellular CA-US HIV RNA by deriving the steady state equation.
