# Supplementary material for: Modeling the Effects of Vorinostat In Vivo Reveals both Transient and Delayed HIV Transcriptional Activation and Minimal Killing of Latently Infected Cells
Source: PLoS Pathog. 2015 Oct 23;11(10):e1005237. doi: 10.1371/journal.ppat.1005237 (PMC4619772; doi:10.1371/journal.ppat.1005237)
Supplement: S9 Fig — Plots show the comparisons of parameter estimates of the rate of CA-US HIV RNA production, α in Log10 (left panel) and the loss rate of sustainably activated cells, d LA (right panel) between the multistage delayed activation model in the main text (‘asymmetric division’) with a model assuming constant proliferation of latently infected cells as in Kim & Perelson [50] (‘Constant proliferation’). In each panel, a dot represents a pair of estimates in one patient, and the dashed lines show the line for y = x. (PDF) [file ppat.1005237.s009.pdf]

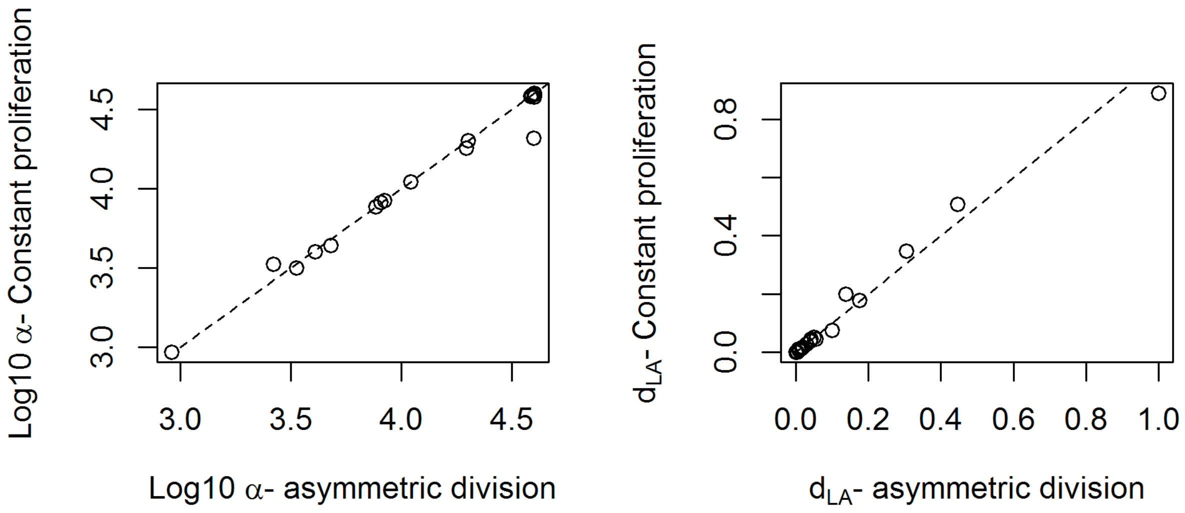

Figure S9. Parameter estimation is robust to changes in the assumption of how latently infected cells are maintained in the absence of vorinostat treatment. Plots show the comparisons of parameter estimates of the rate of CA-US HIV RNA production, α in Log_{10} (left panel) and the loss rate of sustainably activated cells, d_{LA} (right panel) between the multistage delayed activation model in the main text ('asymmetric division') with a model assuming constant proliferation of latently infected cells as in Kim & Perelson (41) ('Constant proliferation'). In each panel, a dot represents a pair of estimates in one patient, and the dashed lines show the line for y=x.
