# Supplementary material for: Modeling the Effects of Vorinostat In Vivo Reveals both Transient and Delayed HIV Transcriptional Activation and Minimal Killing of Latently Infected Cells
Source: PLoS Pathog. 2015 Oct 23;11(10):e1005237. doi: 10.1371/journal.ppat.1005237 (PMC4619772; doi:10.1371/journal.ppat.1005237)
Supplement: S10 Fig — Simulation results using best-fit parameter values (lines) for Patient VOR001 are shown. Diamonds and ‘x’s show the simulation results using parameter values with a 2-fold increase or 2-fold decrease from the best-fit parameters, respectively. (PDF) [file ppat.1005237.s010.pdf]

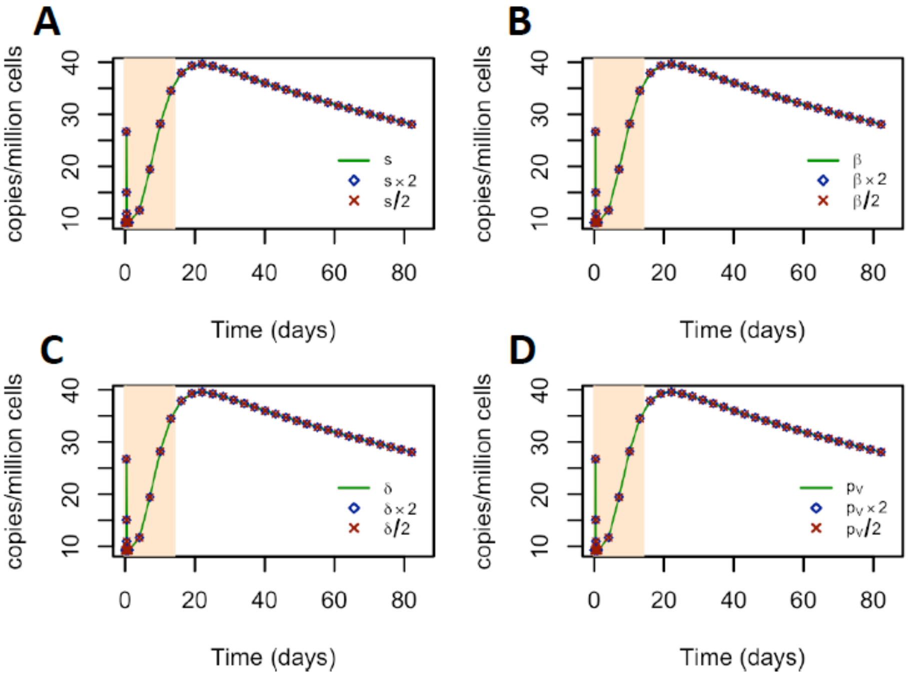


**Figure S10. Sensitivity analysis for changes in the values of fixed parameters governing the dynamics of target cells and infected cells, i.e. *s* (in panel A), $\beta$ (in panel B), $\delta$ (in panel C) and $p_V$ (in panel D).** Simulation results using best-fit parameter values (lines) for Patient VOR001 are shown. Diamonds and 'x' dots show the simulation results using parameter values with a 2-fold increase or 2-fold decrease from the best-fit parameters, respectively. Changing parameter values within this range yields no discernable difference in the dynamics.
