# Supplementary material for: Modeling the Effects of Vorinostat In Vivo Reveals both Transient and Delayed HIV Transcriptional Activation and Minimal Killing of Latently Infected Cells
Source: PLoS Pathog. 2015 Oct 23;11(10):e1005237. doi: 10.1371/journal.ppat.1005237 (PMC4619772; doi:10.1371/journal.ppat.1005237)
Supplement: S1 Table — (PDF) [file ppat.1005237.s011.pdf]

Table S1. Best fit parameter values of the direct activation model to the full dataset in each patient.

Patient	α (copies/ml/day)	d_{LA} (/day)	ν (/day)	RNA_0 (copies/ml)	t_0 (day)
VOR001	39791	0.01	0.00	6.9	0.50
VOR002	1758	0.01	6.58	7.2	0.23
VOR003	6492	0.01	98.09	184.1	0.00
VOR004	2406	1.00	33.56	36.3	0.08
VOR006	1636	0.01	0.28	36.0	0.50
VOR008	34991	0.04	0.04	124.2	0.00
VOR009	6303	0.01	1.55	107.7	0.10
VOR010	6469	0.98	1.15	14.5	0.00
VOR011	37603	0.03	0.00	3.7	0.00
VOR013	27984	0.16	0.01	13.6	0.00
VOR014	39976	0.16	0.03	32.7	0.00
VOR015	39989	0.10	0.38	44.8	0.10
VOR016	39997	0.01	0.04	61.2	0.00
VOR017	39997	0.03	0.01	61.2	0.00
VOR018	33583	0.14	0.02	8.7	0.00
VOR019	1473	0.01	9.78	2.7	0.29
VOR020	13716	0.06	26.10	66.4	0.30
VOR021	39654	0.24	0.31	210.3	0.00
VOR022	13758	1.00	41.92	149.1	0.39
VOR023	15177	0.01	0.20	93.7	0.00
**Mean**	**22138**	**0.05***	**0.43***	**63.2**	**0.12**
**Standard Deviation**	**16296**	**5.15***	**21.52***	**62.6**	**0.18**

* The geometric mean and geometric standard deviation across patients are reported for these parameters, since the estimated values of these parameters vary by several orders of magnitude.
