# Supplementary material for: Modeling the Effects of Vorinostat In Vivo Reveals both Transient and Delayed HIV Transcriptional Activation and Minimal Killing of Latently Infected Cells
Source: PLoS Pathog. 2015 Oct 23;11(10):e1005237. doi: 10.1371/journal.ppat.1005237 (PMC4619772; doi:10.1371/journal.ppat.1005237)
Supplement: S2 Table — (PDF) [file ppat.1005237.s012.pdf]

Table S2. Best fit parameter values of the direct activation model to the data from the first 7-day treatment in each patient.

Patient	α (copies/ml/day)	d_{LA} (/day)	ν (/day)	RNA_0 (copies/ml)	t_0 (day)
VOR001	1003	10.00	37.94	8.3	0.20
VOR002	7407	0.42	0.45	6.9	0.08
VOR003	9869	0.22	21.91	133.4	0.00
VOR004	8029	3.39	4.54	21.8	0.05
VOR006	40000	0.01	0.01	34.5	0.25
VOR008	5649	1.19	99.15	128.7	0.00
VOR009	29548	0.47	0.31	96.6	0.10
VOR010	5600	1.80	1.61	18.1	0.00
VOR011	384	0.13	3.48	2.2	0.00
VOR013	8286	0.70	0.20	11.2	0.25
VOR014	38304	0.69	0.06	32.9	0.00
VOR015	39975	0.01	0.37	35.5	0.10
VOR016	11702	9.87	10.71	105.0	0.03
VOR017	4513	0.03	2.57	48.8	0.00
VOR018	11407	6.85	2.45	14.4	0.10
VOR019	12756	0.73	0.14	4.2	0.22
VOR020	20372	0.08	0.63	58.2	0.11
VOR021	39816	0.17	0.11	324.9	0.02
VOR022	36984	0.63	0.11	114.0	0.00
VOR023	39993	0.18	0.09	96.2	0.00
**Mean**	**18580**	**0.43***	**0.87***	**64.8**	**0.08**
**Standard Deviation**	**15221**	**7.20***	**10.73***	**75.8**	**0.09**

* The geometric mean and geometric standard deviation across patients are reported for these parameters, since the estimated values of these parameters vary by several orders of magnitude.
