# Supplementary material for: Modeling the Effects of Vorinostat In Vivo Reveals both Transient and Delayed HIV Transcriptional Activation and Minimal Killing of Latently Infected Cells
Source: PLoS Pathog. 2015 Oct 23;11(10):e1005237. doi: 10.1371/journal.ppat.1005237 (PMC4619772; doi:10.1371/journal.ppat.1005237)
Supplement: S3 Table — (PDF) [file ppat.1005237.s013.pdf]

Table S3. Best fit parameter values of the delayed activation model to the full data set in each patient.

Patient	α (copies/ml/day)	d_{LA} (/day)	ν (/day)	k_T (/day)	k_W (/day)	RNA_0 (copies/ml)	t_0 (day)
VOR001	1739	0.01	59.02	26.62	0.04	4.1	0.26
VOR002	33850	0.01	0.14	2.31	0.00	7.8	0.10
VOR003	22586	0.02	2.55	1.75	0.02	161.5	0.00
VOR004	8392	0.01	4.28	3.21	0.00	16.7	0.04
VOR006	1493	0.01	1.75	20.94	0.88	35.5	0.50
VOR008	37987	0.07	2.03	7.99	0.04	88.2	0.00
VOR009	39201	0.01	0.31	1.15	0.03	82.1	0.07
VOR010	2930	0.75	38.12	16.17	2.32	16.0	0.08
VOR011	3069	0.05	0.21	3.39	0.05	3.5	0.00
VOR013	10652	0.01	0.10	0.51	0.01	12.1	0.22
VOR014	35839	0.39	0.13	4.20	0.14	32.7	0.00
VOR015	39669	0.17	0.41	0.17	1.89	35.8	0.10
VOR016	33260	0.02	100.00	9.29	0.06	28.8	0.10
VOR017	37937	0.09	0.31	2.45	0.03	50.6	0.00
VOR018	7653	0.06	21.66	3.60	0.02	10.2	0.32
VOR019	39343	0.01	0.04	0.86	0.00	4.7	0.21
VOR020	16826	0.07	35.69	2.67	31.06	58.8	0.30
VOR021	39002	0.26	1.07	1.84	0.30	209.2	0.01
VOR022	34061	0.05	0.20	0.94	0.01	113.2	0.08
VOR023	17347	0.01	0.20	0.10	0.34	93.8	0.00
**Mean**	**23786**	**0.04***	**1.16***	**2.57***	**0.08***	**53.1**	**0.11**
**Standard Deviation**	**14840**	**3.86***	**9.52***	**4.26***	**9.10***	**56.5**	**0.13**

* The geometric mean and geometric standard deviation across patients are reported for these parameters, since the estimated values of these parameters vary by several orders of magnitude.
