# Supplementary material for: Modeling the Effects of Vorinostat In Vivo Reveals both Transient and Delayed HIV Transcriptional Activation and Minimal Killing of Latently Infected Cells
Source: PLoS Pathog. 2015 Oct 23;11(10):e1005237. doi: 10.1371/journal.ppat.1005237 (PMC4619772; doi:10.1371/journal.ppat.1005237)
Supplement: S4 Table — (PDF) [file ppat.1005237.s014.pdf]

Table S4. Best fit parameter values of the multistage delayed activation model to the full data set in each patient.

Patient	n	α (copies/ml/day)	d_{LA} (/day)	ν (/day)	k_T (/day)	k_W (/day)	RNA_0 (copies/ml)	t_0 (day)
VOR001	4	912	0.01	69.55	25.00	0.10	6.9	0.31
VOR002	10	39963	0.04	0.09	1.34	0.03	7.8	0.08
VOR003	10	8051	0.00	100.00	0.18	0.10	141.5	0.06
VOR004	1	8392	0.01	4.28	3.21	0.00	16.7	0.04
VOR006	4	3346	0.02	0.67	0.21	0.04	35.4	0.92
VOR008	4	39739	0.10	1.65	8.70	0.06	108.5	0.00
VOR009	10	39001	0.01	0.24	0.62	0.06	96.2	0.09
VOR010	3	7674	1.00	7.29	15.02	0.91	15.2	0.05
VOR011	6	2627	0.04	1.11	8.66	0.07	3.4	0.06
VOR013	9	4069	0.00	0.14	0.12	0.03	11.0	0.00
VOR014	9	39954	0.45	0.12	3.70	0.14	32.7	0.00
VOR015	2	39951	0.18	0.41	0.17	7.21	45.0	0.15
VOR016	2	38270	0.03	5.16	18.58	0.06	61.3	0.04
VOR017	7	39849	0.05	0.18	0.71	0.05	44.0	0.00
VOR018	1	7653	0.06	21.66	3.60	0.02	10.2	0.32
VOR019	1	39343	0.01	0.04	0.86	0.00	4.7	0.21
VOR020	7	19619	0.06	0.84	0.10	0.03	59.4	0.18
VOR021	10	39902	0.31	0.90	1.02	0.22	204.1	0.00
VOR022	10	4796	0.02	62.63	0.06	0.05	119.8	0.33
VOR023	6	10991	0.00	99.26	2.65	0.50	92.6	0.33
**Mean**		**21705**	**0.04***	**1.81***	**1.29***	**0.08***	**55.8**	**0.16**
**Standard Deviation**		**16978**	**4.01***	**11.53***	**6.16***	**4.44***	**55.1**	**0.21**

* The geometric mean and geometric standard deviation across patients are reported for these parameters, since the estimated values of these parameters vary by several orders of magnitude.
